# Patients with double/triple copy number gains on C-MYC, BCL2, and/or BCL6 treated with standard chemotherapy have a similarly poor prognosis than those with high-grade B cell lymphoma with C-MYC and BCL2 and/or BCL6 rearrangements: a single-center experience on a consecutive cohort of large B cell lymphomas

**DOI:** 10.1007/s00277-020-04124-0

**Published:** 2020-07-01

**Authors:** Ella Willenbacher, Wolfgang Willenbacher, Roman Weger, Wolf Dominik, Claudia Manzl, Andrea Brunner

**Affiliations:** 1 Internal Medicine V - Haematology and Oncology, Medical University of Innsbruck, Innsbruck, Tyrol Austria; 2 ONCOTYROL - Center for personalized Cancer Medicine, Innsbruck, Austria; 3 Institute of Pathology, Neuropathology and Molecular Pathology, Medical University of Innsbruck, Innsbruck, Austria

**Keywords:** HGBL, DLBCL, Double hit, C-MYC, BCL2, BCL6

## Abstract

High-grade B cell lymphomas with rearrangements on C-MYC and BCL2 and/or BCL6 (HGBL with MYC and BCL2 and/or Bcl6 rearrangement) are associated with worse clinical outcomes and thus were introduced as a separate new category in the recently updated WHO classification. From 2012 to 2016, we analyzed a consecutive cohort of large B cell lymphomas (LBCLs) for C-MYC, BCL2, and BCL6 rearrangements and correlated our results with clinical-pathological parameters. Ten of 78 (13%) cases had a C-MYC and BCL2 and/or BCL6 rearrangement, so-called double or triple hit (DH), while double/triple copy number gains (CNGs) were found in eight (10%) patients. Patients with a high-grade lymphoma with DH or CNG progressed significantly more often after first-line chemotherapy (*p* = 0.005). When treated with standard chemotherapy, patients with a DH or CNG had a significantly worse overall (OS) and recurrence free survival (RFS) compared with all other patients (*p* = 0.033 and *p* < 0.001, respectively). Thus, patients with a diffuse large B cell lymphoma, harboring a double/triple CNG, seem to have a similar poor prognosis than those with a DH. Though our data can only be regarded as preliminary, our results warrant further investigations to fully elucidate the role of CNGs as well as underlying molecular mechanisms resulting in aggressive behavior in LBCL.

## Introduction

Large B cell lymphomas (LBCLs) comprise a heterogeneous group of lymphomas. While some of them, such as Burkitt lymphoma, have a well-defined morphology and molecular pathology, others are less well defined and disease outcome is highly variable. Especially, the group of diffuse large B cell lymphomas, not otherwise specified (DLBCL, NOS) consists of different morphological, immunohistochemical, and molecular subgroups and various subtypes significantly differing in clinical behavior and treatment outcome [1]. The WHO classification of hematopoietic tumors from 2008 introduced a provisional entity called large cell B cell lymphoma with features between a DLBCL, NOS and Burkitt lymphoma (BCLU) including large B cell lymphomas with Burkitt-like morphology, but distinct immunohistological features and the frequent presence of C-MYC rearrangements often together with a BCL2 and/or BCL6 rearrangement, so-called double/triple hit (DH) [2, 3]. These cases have been widely discussed in the literature and have been associated with a poor prognosis [4–7]. However, rearrangements of C-MYC together with BCL2 and/or BCL6 have also been reported in otherwise classical DLBCL, NOS [8, 9]. In addition, ill-defined criteria for diagnosing BCLU have prevented a more consistent and comprehensible usage of the diagnosis [10, 11]. Therefore, the current WHO classification includes all B cell lymphomas with rearrangements of C-MYC and BCL2 and/or BLC6 into the category of high-grade B cell lymphomas with rearrangements of C-MYC and BCL2 and/or BLC6 (HGBL with MYC and BCL2 and/or BCL6 rearrangement) irrespective of morphology, while lymphomas without rearrangement, which would otherwise fit into the former category of BCLU, are now summarized as HGBL, NOS [12].

Beside C-MYC rearrangements, expression of C-MYC protein > 40% together with BCL2 expression in > 50% of lymphoma cells has been reported to be associated with a poor outcome which is however better than in HGBL, C-MYC. Therefore, overexpression is only seen as a prognostic factor [9, 12]. In addition, several studies point towards a role of copy number gains (CNG) on C-MYC and/or BCL2 and/or BCL6 [13–15]. In fact, patients with CNG are reported to have a similarly poor prognosis than those with HGBL with MYC and BCL2 and/or BCL6. [13–15].

There is currently no agreement if an analysis for a C-MYC, BLC2, and BCL6 rearrangement should be done in every DLBCL, NOS upon diagnosis or should be restricted to cases with GCB phenotype, a high-grade morphology, or C-MYC positivity [12].

From 2012 to 2016, we analyzed a consecutive cohort of DLBCL, NOS for the presence of C-MYC, BCL2, and BCL6 rearrangements and correlated our results with clinical-pathological parameters.

## Material and methods

### Patients

From 2012 to 2016, all LBCLs diagnosed at the Institute of Pathology, Neuropathology, and Molecular Pathology, Medical University of Innsbruck, were upon diagnosis routinely analyzed for the presence of rearrangements on C-MYC, as well as BCL2 and BCL6 by means of fluorescence in situ hybridization (FISH), irrespective if they were de novo lymphomas or transformed from an underlying small cell lymphoma. Specimens included biopsies from lymph nodes, extra nodal sites, and bone. The study was conducted according to the ICH-GCP guidelines and the declaration of Helsinki. Ethical approval was obtained from the ethical committee of the Medical University of Innsbruck, and all patients gave an informed consent for study participation (EK-Nr.1213/2017).

### Morphology and immunohistochemistry

Immunohistochemistry and EBER in situ hybridization were done upon diagnosis using an automated platform (Benchmark ULTRA, Ventana Medical Systems, Tucson, USA). The following antibodies were routinely applied: CD20 (clone L26, prediluted, Ventana Medical Systems, Tucson, USA), CD79 (clone SP19, prediluted, Ventana Medical Systems, Tucson, USA), CD5 (clone SP19, prediluted, Ventana Medical Systems, Tucson, USA), Ki67 (clone Mib-1, dilution 1:100, DAKO, Leiden, the Netherlands), CD10 (clone SP67 prediluted, Ventana Medical Systems, Tucson, USA), BCL2 (clone 124, prediluted, Ventana Medical Systems, Tucson, USA), BCL6 (clone GI191E/A8, prediluted, Ventana Medical Systems, Tucson, USA), MUM1 (clone MRQ 43, prediluted, Ventana Medical Systems, Tucson, USA), C-MYC (clone Y69, prediluted, Ventana Medical Systems, Tucson, USA), and INFORM EBER (Epstein-Barr virus early RNA) Probe (Cat. Nr. 800-2842, Ventana Medical Systems, Tucson, USA).

All LBCLs were classified according to the WHO classification of tumors of the hematopoietic and lymphoid tissues (2008) and in addition were subdivided into germinal center (GCB) and non-germinal center (non-GCB) subtypes [2, 16]. A cutoff of 40% C-MYC and 50% BCL2 positive lymphoma cells was used to identify double expressors [9, 12].

### Fluorescence in situ hybridization

FISH for C-MYC, BCL2, and BCL6 was performed using dual color break apart probes for each locus (Z-290-200 ZytoLight SPEC MYC Dual Color Break Apart Probe, Z-2192-200 ZytoLight SPEC BCL2 Dual Color Break Apart Probe, and Z-2177-200 ZytoLight SPEC BCL6 Dual Color Break Apart probe; Zytovision, Bremerhaven, Germany). Tissue was mounted in a DAPI containing mounting media (Zytovision, Bremerhaven, Germany), and interphase nuclei were monitored using a fluorescence microscope (Axioplan 2, Zeiss, Oberkochen, Germany) equipped with a 63× oil objective.

For evaluation, at least 100 non-overlapping nuclei with sufficient bright signals in at least 3 areas had to be evaluated. CNGs were diagnosed when there were ≥ 3 fusion signals/cell. We did not distinguish between high (> 5 fusion signals) and low amplifications because current literature did not report any difference in survival between high and low copy numbers [13]. The cutoffs for considering a tumor sample positive for MYC, BCL2, and BCL6 by FISH were determined by assessing 10 normal lymph nodes (100 nuclei per sample) and choosing cutoffs of three standard deviations above the mean. The cutoffs were low for both split signals and CNGs (≤ 3%), but cases involved in the study in general had abnormalities (split signals, CNGs) in ≥ 10% of nuclei. Therefore, we considered all cases with ≤ 10% (and ≥ 3%) abnormalities with caution and increased the number of nuclei counted or repeated the FISH analysis, if necessary. Ultimately, only one case out of 78 (1.2%), which was then regarded as negative, had to be repeated.

### Clinical characteristics and treatment

Clinical data, such as age, sex, Ann Arbor stage, International Prognostic Index (IPI), and lactate dehydrogenase (LDH) levels at diagnosis, as well as first-line treatment and response to treatment were retrospectively collected from patients’ files at the University Clinics of Internal Medicine V, Hematology & Oncology. According to Cheson et al., response criteria were defined as complete response confirmed (CRc), complete response unconfirmed (CRu), partial response (PR), stable disease (SD), and progressive disease (PD). Results of the FISH analysis did not influence the choice of treatment [17].

### Statistical analysis

The Statistical Package of Social Sciences (SPSS 26.0 for Windows) was used. The *χ*^2^ test was used to test the relationship between HGL-DH, DLBCL-CNG, and DLBCL without DH-CNG and clinical as well as pathological parameters. Differences concerning overall survival and recurrence free survival between HGL-DH, DLBCL-CNG, and DLBCL without DH-CNG were analyzed by the Kaplan-Meier method and compared by the log rank test. A multivariate analysis was performed to identify independent prognostic markers for OS and RFS using a Cox multistep regression model. A *p* value < 0.05 was considered significant.

## Results

### Patients

Our study cohort finally included 78 patients (44 men and 34 women) with a mean age of 62.47 years, range from 25 to 93) with a diagnosis of DLBCL, NOS, or BCLU. Other specific types of aggressive lymphomas such as Burkitt lymphoma, primary mediastinal large B cell lymphoma, EBV^+^ DLBCL, or plasmablastic lymphoma were excluded upon diagnosis. According to the WHO 2008, of the 78 patients, 76 (97.5%) were classified as DLBCL, NOS including four patients with a known history of underlying small cell lymphoma (one chronic lymphocytic leukemia and three follicular lymphomas) and two (2.5%) patients were diagnosed as BCLU. Three lymphomas were diagnosed as monomorphic B cell post-transplant lymphoproliferative disease (monomorphic B cell PTLD) fulfilling the criteria of DLBCL: one EBV positive and two EBV negative. One DLBCL was HIV associated and EBV positive. Thirty-three cases were initially diagnosed in a lymph node while 45 were diagnosed in an extra nodal site such as the liver and soft tissue and bone.

### Fluorescence in situ hybridization and reclassification

FISH results were available for BCL2 in 76/78 (97%), for BCL6 in 70/78 (90%), and for C-MYC in 77/78 (99%) patients. Lack of data was due to limited material. Six DLBCL, NOS, and one BCLU had a “double hit” (four with C-MYC/BCL2 and two with C-MYC/BCL6) while two DLBCL, NOS and one BCLU presented with a “triple hit”; thus, these 10 (13%) cases would now most likely have been classified into the category of HGBL with MYC and BCL2 and/or BCL6 rearrangement.

Rearrangements on either C-MYC or BCL2 or BCL6 were found in three (4%), eight (10%), and 10 (13%) cases of DLBCL, NOS. CNGs on C-MYC and BCL2 and/or BCL6 were present in eight (10%) cases. In three cases with double/triple CNG an additional BCL2 rearrangement and in two an additional BCL6 rearrangement were detected. In addition, it should be noted that no rearrangement or double/triple CNG was found in all three PTLDs: the transformed CLL, a transformed FL, and the HIV+ DLBCL. One secondary DLBCL, NOS arising from a FL had a DH, while in one transformed lymphoma, FISH could not be evaluated and the case was therefore excluded from analysis. Patient’s clinical and pathological characteristics are summarized in detail in Table 1. For statistical analysis, the 10 (13%) cases with a DH were summarized as high-grade double hit lymphoma (HGL-DH), the 8 (10%) cases with double/triple CNG as DLBCL-CNG (taken together as LBCL-DH-CNG), and the remaining 60 cases as DLBCL without DH-CNG.

**Table 1:** Clinical and pathological characteristics of the 78 patients

		HGL-DH (*N* = 10)	DLBCL-CNG (*N* = 8)	DLBCL without DH-CNG (*N* = 60)	*p* value
Age	< 65	6 (60%)	3 (38%)	30 (50%)	0.59
	> 65	4 (40%)	5 (62%)	30 (50%)	
Sex	Male	4 (40%)	6 (75%)	34 (57%)	0.39
	Female	6 (60%)	2 (15%)	26 (43%)	
Stage	I-II	2 (20%)	0 (0%)	17 (28%)	0.30
	III-IV	6 (60%)	7 (88%)	37 (62%)	
	Unknown	2 (20%)	1 (12%)	6 (10%)	
LDH	Elevated	6 (60%)	6 (75%)	28 (47%)	0.15
IPI Score	Low	1 (10%)	1 (12.5%)	13 (21%)	0.49
	Low-intermediate	1 (10%)	4 (50%)	13 (21%)	
	High-intermediate	3 (30%)	1 (12.5%)	15 (25%)	
	High	3 (30%)	1 (12.5%)	14 (23%)	
	Unknown	2 (20%)	1 (12.5%)	6 (10%)	
Localization	Nodal	6 (60%)	3 (38%)	24 (40%)	0.63
	Extranodal	4 (40%)	5 (62%)	36 (60%)	
Immunohistochemistry	BCL2 > 50% +	7 (70%)	8 (100%)	34 (57%)	0.09
	BCL6 +	7 (70%)	5 (62%)	35 (58%)	0.77
	CD10 +	9 (90%)	3 (38%)	26 (43%)	0.02
	MUM1 +	2 (20%)	6 (75%)	35 (58%)	0.06
	C-MYC > 40% +*	5 (100%)	0 (0%)	12 (22%)	< 0.001
Double expressors	Yes	5 (50%)	0 (0%)	7 (10%)	< 0.001
	No	0 (0%)	6 (75%)	41 (68%)	
	Unknown	5 (50%)	2 (25%)	12 (22%)	
Hans classifier	GCB	9 (90%)	3 (38%)	29 (48%)	0.05
	Non-GCB	1(100%)	5 (62%)	30 (50%)	
	Unknown	0 (0%)	0 (0%)	1 (1.5%)	
Ki67	Mean (%)	85.50	83.13	78.6	0.41
	Median (%)	90	87.5	80	
	< 90	4 (40%)	4 (50%)	37 (61%)	0.29
	≥ 90	6 (60%)	4 (50%)	22 (36%)	
Rearrangement	BCL2 +	8 (80%)	3 (38%)	5 (12%)	< 0.001
	BCL6 +	5 (50%)	2 (25%)	8 (15%)	< 0.001
	C-MYC +	10 (100%)	0 (0%)	3 (6%)	< 0.001
	Double/triple CNG +	0 (0%)	8 (100%)	0 (0%)	< 0.001

*N* number of cases, *LDH* lactate dehydrogenase

*Only available in 59 cases including 5 HGL-DH

### Pathological characteristics

Of the 78 patients, 41 were classified as GCB and 36 as non-GCB subtype, while in one case, the immunohistochemical subtype could not be determined due to limited tissue. The majority of HGL-DH was of GCB subtype; however, one case was classified as non-GCB subtype. Expression of C-MYC was available in 59/78 (76%) cases, including 5/10 (50%) HGL-DH as well as 5/8 (62%) DLBCL-CNG. BCL2 immunohistochemistry was done in 77/78 (99%) cases. C-MYC expression > 40% and BCL2 expression in > 50% of lymphoma cells were detected altogether in 12/58 (21%) cases. However, only six patients (10%) were true “double expressors” while one case had a C-MYC rearrangement only and the other five (8%) cases belonged to the HGL-DH group. All five DLBCL-CNGs, where C-MYC immunohistochemistry was available, showed a C-MYC expression < 40%. Also, DLBCL-CNG compared to HGL-DH and DLBCL without DH-CNG had a significantly lower expression of CD10 (*p*^chi-square^ = 0.02), a higher expression of BCL2 and MUM1 (*p*^chi-square^ = 0.09 and < 0.001, respectively) and thus significantly more often belonged to the non-GCB subgroup (*p*^chi-square^ = 0.05; see Table 1).

### Treatment and disease response

Information on treatment was available in 70/78 (90%) and on disease response in 59/78 (76%) patients. While the vast majority of patients received standard treatment, four (5%) patients had a palliative treatment and four (5%) patients did not receive any therapy, mainly due to very old age (> 90 years) and co-morbidities. The standard treatment consisted of R-CHOP (42 patients) and CHOP-like therapy (R-COMP in 14 patients and R^2^-CHOP in two patients) as well as escalated regimens in 4 patients (GMALL, R-DA-EPOCH). Of eight (10%) patients, no information on first-line treatment was available. While in DLBCL without DH-CNG, CR was achieved in nearly half (47%) of the patients; 6/10 (60%) patients with HGL-DH and 3/8 (38%) patients with DLBCL-CNG had PD after initial treatment (*p*^chi-square^ = 0.005; see Table 2).

**Table 2:** Treatment and response of the 78 patients in the study

		HGL-DH (*N* = 10)	DLBLC-CNG (*N* = 8)	DLBCL without DH-CNG (*N* = 60)	*p* value
First-line treatment	Standard	8 (80%)	5 (62.5%)	49 (82%)	0.52
	Palliative	0 (0%)	0 (0%)	4 (66%)	
	None	1 (10%)	1 (12.5%)	2 (3%)	
	Unknown	1 (10%)	2 (25%)	5 (8%)	
Autologous Tx	Yes	2 (20%)	1 (12.5%)	6 (10%)	0.28
	No	6 (60%)	4 (50%)	47 (78%)	
	Unknown	2 (20%)	3 (37.5)	7 (12%)	
Initial response	CRc/CRu	3 (30%)	2 (25%)	30 (47%)	0.005
	PR/SD	0 (0%)	0 (0%)	9 (15%)	
	PD	6 (60%)	3 (38%)	6 (10%)	
	Unknown	1 (10%)	3 (38%)	15 (25%)	
Recurrence	Yes	5 (50%)	3 (38%)	11 (18%)	0.18
	No	4 (40%)	5 (62%)	42 (70%)	
	Unknown	1 (10%)	0 (0%)	7 (12%)	
Survival	Alive	6 (60%)	4 (50%)	17 (28%)	0.14
	Dead	3 (30%)	2 (25%)	36 (60%)	
	Unknown	1 (10%)	2 (25%)	7 (12%)	

*N* number of cases, Tx transplantation

### Overall survival

**Fig. 1:**
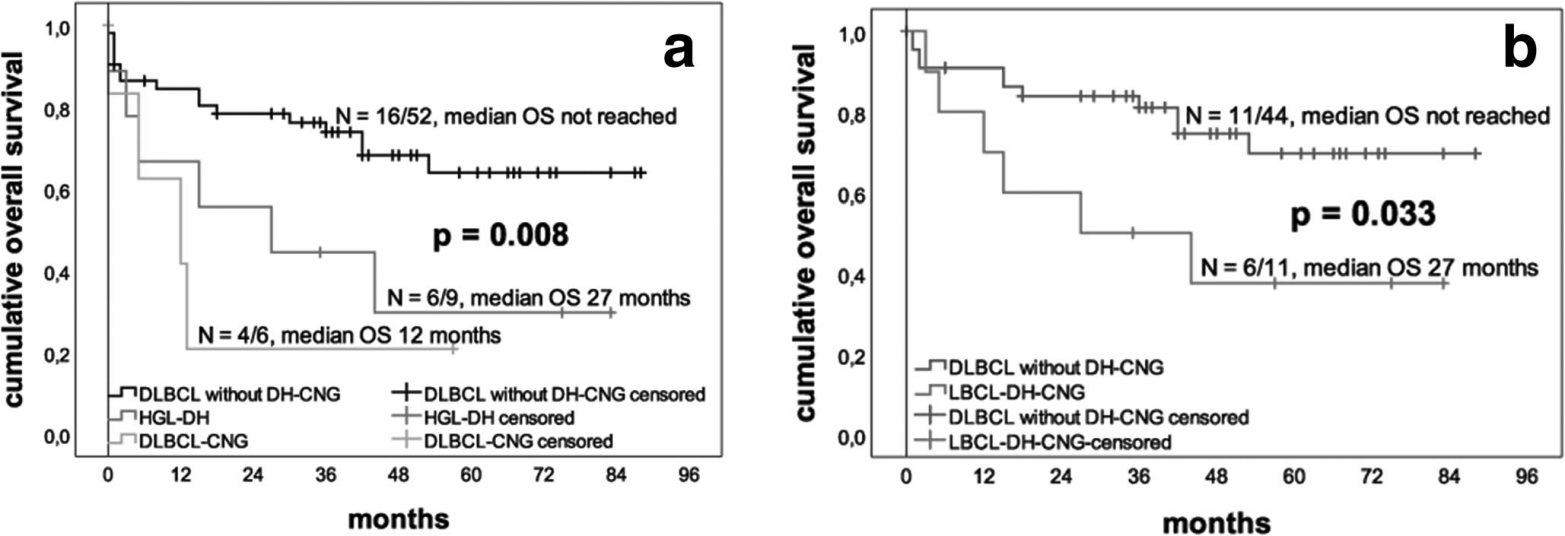
Patients with HGL-DH and DLBCL-CNG have a significantly worse OS than those with DLBCL without DH-CNG (a); treated with standard chemotherapy, patients with LBCL-DH-CNG have a significantly worse OS than all other patients with DLBCL without DH-CNG (b) (*N* = number of events/number of cases)

Patients with DLBCL-CNG and HGL-DH had a significantly worse OS than those with DLBCL without DH-CNG (4/6 (66%) DLBCL-CNG, median OS 12 months versus 6/9 (66%) HGL-DH, median OS 27 months versus 16/52 (31%) DLBCL without DH-CNG, median OS not reached; *p*^log rank^ = 0.008; see Fig. 1a). When comparing only patients with HGL-DH and all other DLBCL, NOS (including also DLBCL-CNG) data only showed a tendency for worse OS for HGL-DH (*p* = 0.09). Taken together, in all patients with LBCL with C-MYC, BCL2, and BCL6 alterations (LBCL-DH-CNG), a worse OS was observed compared to DLBCL without DH-CNG (*p*^log rank^ = 0.004).

When the analysis was restricted to patients, who received standard treatment, patients with LBCL-DH-CNG still had a worse OS than those with DLBCL without DH-CNG (6/11 (54.5%) LBCL-DH-CNG, median OS 27 months versus 11/44 (25%) DLBCL without DH-CNG, median OS not reached; *p*^log rank^ = 0.033).

Stratified for age, stage, LDH at diagnosis, IPI, risk stratification, extra nodal localization of disease Ki67 and cell of origin subtype (GCB versus non-GCB) age > 65 years (*p*^log rank^ = 0.015), stage III-IV (*p*^log rank^ = 0.037), GCB subtype (*p*^log rank^ = 0.019), extra nodal localization (*p*^log rank^ < 0.001), and Ki67 > 90% (*p*^log rank^ = 0.04) were significantly associated with a worse OS for patients with LBCL-DH-CNG treated with standard chemotherapy.

Beside DH-CNG, the only other factors significantly influencing OS in patients treated with standard therapy was stage (*p*^log rank^ = 0.046), while IPI and risk stratification only showed a tendency for worse OS for high IPI (4–5) and high intermediate/high risk lymphomas (*p*^log rank^ = 0.149 and 0.073, respectively).

Multivariate analysis including DH-CNG status, stage, IPI, and risk stratification showed that none is an independent prognostic factor for worse OS in patients treated with standard chemotherapy.

### Recurrence free survival

**Fig. 2:**
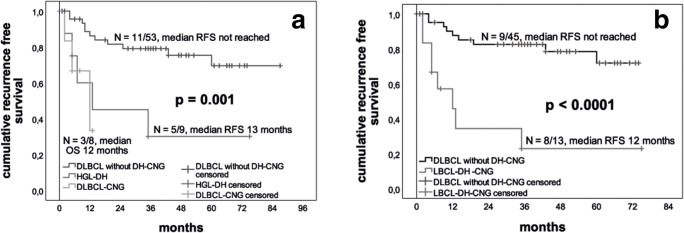
Patients with HGL-DH and DLBCL-CNG have a significantly worse RFS than those with DLBCL without DH-CNG (a); treated with standard chemotherapy, patients with LBCL-DH-CNG have a significantly worse RFS than all patients with DLBC without DH-CNG (b) (*N* = number of events/number of cases)

Patients with HGL-DH had a significantly worse recurrence free survival than those with DLBCL, NOS (including DLBCL-CNG) (*p*^log rank^ = 0.013). DLBCL-CNGs were also associated with significantly shorter RFS similar to patients with HGL-DH (3/8 (38%) DLBCL-CNG, median RFS 12 months versus 5/9 (55%) HGL-DH, median RFS 13, months versus 11/53 (21%) DLBCL without DH-CNG, median RFS not reached; *p*^log rank^ = 0.001; see Fig. 2a). Taken together, all patients with LBCL-DH-CNG had a worse RFS compared to DLBCL without DH-CNG (*p*^log rank^ < 0.001).

This also holds true when analysis was restricted to patients receiving standard treatment (8/13 (61.5%) LBCL-DH-CNG, median RFS 12 months versus 9/45 (20%) DLBCL without DH-CNG, median RFS not reached; *p*^log rank^ < 0.0001; see Fig. 2b).

Stratified for age, stage, LDH at diagnosis, IPI, risk stratification, localization of disease, and Ki67 proliferation index, a significantly worse RFS was observed in LBCL-DH-CNG patients with age > 65 years (*p*^log rank^ < 0.0001), stage III-IV (*p*^log rank^ < 0.0001), IPI 0–3 (*p*^log rank^ < 0.001), pathologic LDH at diagnosis (*p*^log rank^ = 0.001), and Ki67 > 90% (*p*^log rank^ = 0.001).

Neither age nor stage, IPI, risk stratification, LDH at diagnosis, localization of disease, and Ki67 proliferation index alone significantly influenced RFS.

## Discussion

In our consecutive cohort of DLBCL, NOS, we identified 10 (12%) HGL-DH and three (4%) DLBCL, NOS with C-MYC rearrangement alone. Our data are in line with previous reports on the frequency of this type of lymphomas in a consecutive cohort, which range from 3 to 21% depending upon the composition of patient population [5]. Double/triple CNGs were found in 10% of patients, where recent reports suggest a frequency of 3.8 to 6% of DLBCL, NOS [13, 15]. The slightly higher number in our group may be due to the analysis of C-MYC, BCL2, as well as BCL6 in virtually all cases, which is seldom done in routine diagnostics.

Our data on HGL-DH once more confirm the need for a different therapeutic approach in patients with LBCL harboring a C-MYC and BCL2 and/or BCL6 rearrangement, though none of the suggested escalated regimens, such as DA-EPOCH-R (dose-adjusted etoposide, prednisolone, vincristine, cyclophosphamide, doxorubicin, and rituximab) or R-CHOP-ibrutinib, proved to be superior in a randomized phase III setting so far [6, 18–23]. As previously reported by other authors, in our study, cases with double/triple CNG had a similarly poor prognosis than HGL-DH [13–15]. Therefore, although the group of HGL-DH includes lymphomas with a defined aberration, this group does not suit all those LBCL with a poor prognosis. Thus, the LBCLs with MYC alterations are not a homogeneous group. In fact, while HGL-DH cases usually show overexpression of C-MYC and GCB subtypes, cases with double/triple CNG lack C-MYC expression > 40% and more often belong to the ABC-group, implicating that the mechanisms by which C-MYC and BCL2 and/or BCL6 alterations finally influence prognosis may be different ones [13, 14]. This is also supported by recent genetic data on DLBCL, NOS which show, depending on the method of analysis, up to five genetic subtypes of DLBCL, NOS, of which C-MYC rearranged or double/triple hit cases were usually part of but not exclusively formed the group of molecular high-grade lymphomas [24–27]. Therefore, further investigations are needed to fully decrypt the molecular basis for a more aggressive behavior in order to delimit this group of patients from all others.

Our study has some limitations. First of all, it is based upon a rather small and heterogeneous group of patients including also cases of known secondary DLBCL, NOS, though the majority of these clinically usual more aggressive lymphomas belonged to the group of DLBCL without CNG. Second, it represents the experience of only a single center; thus, to further support our results, a larger prospective and multicenter trial would be necessary. Third, due to the small numbers of DH-CNG cases, a further subdivision (i.e., low versus high amplification; amplification with/or without additional rearrangement on either C-MYC or BLC2 or BCL6) could not be performed. Also, since the data were collected retrospectively, information upon polyploidy was not available for routine FISH and does not include centromere probes, but break apart probes for each specific locus.

According to the actual WHO, reporting double/triple CNG is not a mandatory component for the diagnosis of a DLBCL, NOS, but given the recent reports on clinical outcome, we suggest that double/triple CNG should be reported similar to overexpression of C-MYC and BCL2 [13–15]. This would require FISH at least for C-MYC rearrangement in any newly diagnosed DLBCL, NOS.

To summarize, though one cannot draw any definite conclusions from our data by now, we believe our results warrant further investigations in larger sample sets and preferably a prospective fashion to fully elucidate the role of CNGs as well as the underlying molecular mechanisms resulting in aggressive behavior in LBCL.
